# Developmental changes in and the relationship between psychological resilience and mental health problems in adolescents relocated for poverty alleviation in the context of COVID-19 epidemic prevention and control

**DOI:** 10.3389/fpubh.2023.1118535

**Published:** 2023-03-21

**Authors:** Hai Zhu, Juan Song, Rong Zhang, Benbin Wang, Xiaosong Shen

**Affiliations:** ^1^School of Teacher Education, Zunyi Normal University, Zunyi, China; ^2^School of Psychology, Guizhou Normal University, Guiyang, China; ^3^Education and Sports Bureau of Zunyi City, Zunyi, China; ^4^Beijing Mental Data Matrix Technology Co., Ltd., Beijing, China

**Keywords:** adolescents relocated for poverty alleviation, psychological resilience, mental health problems, latent growth model, cross-lagged regression analysis

## Abstract

**Background:**

Adolescents relocated for poverty alleviation have considerable mental health problems (MHPs) in the context of changing living environments and COVID-19 epidemic prevention and control, and psychological resilience (PR) is closely related to MHPs. Existing studies have mainly used cross-sectional research to investigate the relationship between PR and MHPs using PR as a predictor variable.

**Objective:**

This study investigated developmental changes in the PR and MHPs of relocated adolescents as well as the relationship between these factors.

**Methods:**

A longitudinal study was conducted to assess the PR and MHPs of 1,284 relocated adolescents. Data were collected at approximately 12-month intervals at three time points: spring of 2020 (T1), spring of 2021 (T2), and spring of 2022 (T3). The 1284 adolescents consisted of 620 males and 664 females; 787 were in the fourth grade of elementary school, 455 were in the first grade of middle school, and 42 were in the first grade of high school. The collected data were analyzed using SPSS 25.0 and Mplus 8.1 and methods such as latent growth models and cross-lagged regression analysis.

**Results:**

(1) The PR level of relocated adolescents showed an overall increasing trend (slope = 0.16, *p* < 0.01), while the MHPs showed an overall decreasing trend (slope = −0.03, *p* < 0.01). (2) The initial PR level differed significantly from the initial MHPs level (β = −0.755, *p* = 0.00), and the rate of change in PR differed significantly from the rate of change in MHPs (β = −0.566, *p* = 0). The initial MHPs level differed significantly from that of PR (β = −0.732, *p* = 0.00), and the rate of change in MHPs differed significantly from the rate of change in PR (β = −0.514, *p* = 0.00). (3) Among the three sets of measurements of PR and MHPs, there were significant pairwise differences.

**Conclusion:**

(1) The PR level of relocated adolescents increased over time, and the MHPs of relocated adolescents decreased over time. (2) The initial PR level of relocated adolescents had a negative predictive effect on the initial MHPs level, and the rate of change in PR had a negative predictive effect on the rate of change in MHPs. (3) The PR and MHPs of relocated adolescents exhibited a bidirectional, mutually influencing relationship.

## 1. Introduction

The mental health of adolescents is inextricably linked to social development. Therefore, adolescent mental health problems (MHPs) have gradually become a focus of heated discussion in society. Adolescents are in a critical period of life development, and the stresses of life, education, and family may lead to the occurrence of psychological problems such as insomnia and anxiety (1, 2). The occurrence of psychological problems is associated with psychological resilience (PR). Studies have shown that PR is significantly positively correlated with positive mental health levels (3, 4) and significantly negatively correlated with MHPs (4, 5). During the COVID-19 pandemic, MHPs among adolescents have been increasing (6), and PR is considered to have a buffering effect in coping with the pressure induced by the pandemic (7, 8). This effect is often achieved through cultivating adolescents' PR to reduce the impact of pandemic-related pressure (9). “Relocation for poverty alleviation” was a measure in China's fight against poverty and occurred during the 2016–2020 period. In other countries, this concept is commonly referred to as “ecological migration” (10). Its main purpose is to help people living in areas with poor natural environments to relocate to resettlement areas, improve their living conditions, promote economic growth, and eventually achieve common prosperity. Adolescents who relocated with their parents to live in resettlement areas may have MHPs due to changes in their living and learning environments (11, 12). With the emergence and continued influence of the COVID-19 epidemic, adolescents are prone to MHPs such as anxiety, depression, and insomnia (13). Adolescents who were relocated for poverty alleviation (hereinafter referred to as “relocated adolescents”) are affected by both changes in living environments and the COVID-19 epidemic and hence have few opportunities for external communication, potentially causing MHPs. The aim of this study was to investigate the developmental changes in and relationship between the PR and MHPs of relocated adolescents in the context of the normalization of epidemic prevention and control.

Resilience is the process by which an individual recovers, maintains, or improves their mental health in the face of adversity (14). Thinking of resilience as a process means that resilience is related to not only the individual, but also the ecosystem, taking into account the impact of the social environment (15). PR reflects a continuous flexible and adaptive response model through which people can cope with changing environmental demands (16). From a longitudinal developmental perspective, PR has variability and plasticity; it can evolve over time (17) and can also improve through interventions and training (18, 19). PR is specific to the background and content, and the process of resilience changes with the population, background and risk exposure (20, 21). Previous studies based on PR under general ecological migration have shown that some immigrant adolescents are resilient despite facing more stress than their local peers (22). At present, it is not clear whether the PR development of adolescents in the context of poverty alleviation and relocation is consistent with the results of general ecological migration.

Traditional clinical psychologists have studied MHPs in adolescents from a pathological perspective, focusing on mental illness, general mental health disorders, emotional and behavioral problems, psychological distress, and disease symptoms (23). Studies have shown that individuals' mental health is related to their social-ecological environment, family education, and individual psychological traits; for example, the on-going COVID-19 epidemic has increased the risk of MHPs in adolescents and has impacted their emotional state and subjective well-being (24). Being a member of a family with a low economic status has a significant positive predictive effect on an individual's MHPs (25). After relocation, the family, school, and social environments of adolescents change substantially, and their economic status contrasts significantly with the surrounding social environment. During this process, they may develop strong psychological discomfort, which manifests as increased levels of depression and loneliness (12). Furthermore, they may also display more problematic behaviors (26). Good family functioning can effectively alleviate the negative emotions and develop good behavioral habits of adolescents (12). In general, the mental health status of relocated adolescents will be affected. An environment of poverty and inequality increases the risk of developing MHPs (27), but the living conditions and environment of individuals are greatly improved due to the support of relocation policies based on poverty alleviation in relocation sites. Therefore, it is of unique value to understand the development of MHPs among relocated adolescents.

Numerous studies have explored the relationship between PR and MHPs, and most have used PR as a predictor of MHPs, such as the influence of PR on anxiety, tension, depression, and other symptoms (28, 29). Adolescents with higher levels of resilience exhibit fewer MHPs (30), that is, PR negatively predicts MHPs. However, some studies have shown that PR and MHPs can be predicators of each other-i.e., there is a bidirectional, mutually influencing relationship between these factors (31, 32). Based on a sample of Chinese college students, Wu et al. (31) found that an individual's PR can significantly predict mental health status in the short-term. Furthermore, the predictive effect of mental health on PR is significant in both the short-term and long-term, indicating a relationship between the two (31). In the case of adolescents in China, the study found that resilience before COVID-19 was a significant negative predictor of mental health problems during the outbreak, and PR and MHPs before the outbreak were positively predictive of PR and MHPs during the outbreak, respectively (32). There has been no study on the two-way relationship between PR and MHPs in adolescents. In the context of the COVID-19 epidemic prevention and control, adolescents relocated for poverty alleviation to live and study in new places may suffer from mental health problems such as loneliness and depression due to poor adaptation (12). Under these dual pressures, relocated individuals may have more prominent mental health problems. In addition, a large number of studies have indicated that adolescents in the context of COVID-19 epidemic prevention and control have a high PR level (33, 34), and the incidence of mental health problems is influenced by PR's positive psychological quality. Additionally, the PR and MHPs of relocated adolescents may have a bidirectional, mutually influencing relationship.

This study aims to investigate, through three follow-up tests, the developmental changes in and the relationship between the PR and MHPs of relocated adolescents in the context of the COVID-19 epidemic prevention and control. First, the developmental trajectories of PR and MHPs are examined. As previously stated, PR and MHPs change over time, leading to Hypothesis 1: The PR level of relocated adolescents increases over time, and the MHPs of relocated adolescents decrease over time. Second, the relationship between PR and MHPs is investigated. On the basis of previous correlation and prospective studies on PR and MHPs, Hypothesis 2 is proposed: The initial PR level of relocated adolescents has a negative predictive effect on the initial level of MHPs, and the rate of change in PR has a negative predictive effect on the rate of change in MHPs. Finally, cross-lagged regression analysis is used to verify the temporal order of the relationship between PR and MHPs. On the basis of the previous elaboration on the interaction between PR and MHPs, we propose Hypothesis 3: There is a bidirectional, mutually influencing relationship between the PR and MHPs of relocated adolescents.

## 2. Methods

### 2.1. Participants

The participants were from two elementary schools, two middle schools, and one high school in Zunyi City, Guizhou Province, China. The survey was organized by the local education department to assess the mental health needs related to the COVID-19 epidemic and to guide the further development of effective strategies to prevent and address MHPs. The participants in this study were 1,450 relocated adolescents. The eligible criteria for the relocated adolescents: (1) They have moved from the original harsh environment to the current environment due to the “Relocation for poverty alleviation”. (2) They moved in 2018. Since there are relocated students from 2017 to 2019, in order to better consider the characteristics of relocated students at the same time, this study selects students who moved into the current living environment in 2018. (3) They are aged between 10 and 19 years old. Among the 1,450 students, 1,284 students completed the surveys at all three time points; 620 were male, and 664 were female, with 787 in the fourth grade of elementary school, 455 in the first grade of middle school, and 42 in the first grade of high school. There was no significant difference between the lost sample and the valid sample in MHP T1 [*t* = −1.646, *p* > 0.05], MHP T2 [*t* = −1.488, *p* > 0.05], MHP T3 [*t* = −1.505, *p* > 0.05], PR T1 [*t* = 0.301, *p* > 0.05], PR T2 [*t* = 1.882, *p* > 0.05], PR T3 [*t* = 1.558, *p* > 0.05], sex [*t* = −1.292, *p* > 0.05], grade [*t* = −0.935, *p* > 0.05], family economic status [*t* = 0.369, *p* > 0.05] and the way of study [*t* = −1.577, *p* > 0.05]. Only data from subjects who participated in all three data collection sessions were selected for this study, and there were no missing values in these data.

### 2.2. Procedure

First, the study was done with the support of the researchers. Second, the survey was conducted in the school's computer room with the consent of the teenager's parents or guardian and was conducted by the local education department, with the help of researchers, to carry out the research smoothly. Finally, data were collected at approximately 12-month intervals at three time points: the first time point in the spring of 2020 (T1), the second time point in the spring of 2021 (T2), and the third time point in the spring of 2022 (T3). At each time point, students were encouraged to complete a series of online questionnaires, including self-reported PR and mental health questionnaires. This study was approved by the Ethics Review Committee of the State Key Laboratory of Cognitive Neuroscience and Learning at Beijing Normal University.

### 2.3. Instruments

#### 2.3.1. PR scale for adolescents

This scale was adapted from the Resilience Scale for Chinese Adolescents (RSCA) (35). The scale includes five factors: goal planning, affect control, positive thinking, family support, and help-seeking. The adapted scale has a total of 18 items, including nine reverse scoring items. The scale is scored on a five-point scale (1 = totally disagree, 2 = relatively disagree, 3 = fairly disagree, 4 = relatively agree, and 5 = totally agree), with higher scores indicating higher levels of PR. Subjects' scores on all items in this study were subsequently averaged, and the internal consistency of each measurement was acceptable (Cronbach's α = 0.852 to 0.892).

#### 2.3.2. Mental health scale for adolescents

This scale was adapted from the Mental Health Scale for Middle School Students in China (MSSMHS) (36) and the Symptom Checklist (SCL-90) (37) and can be used to assess the mental health of primary and secondary school students. The scale includes five dimensions, namely, interpersonal sensitivity, hostility, anxiety, compulsion, and paranoia. The adapted scale has a total of 27 items and is scored on a five-point scale (1 = none, 2 = mild, 3 = moderate, 4 = relatively severe, and 5 = severe). Higher scores on the scale indicate more severe MHPs in individuals. It can not only measure the overall mental health status of respondents but also evaluate respondents' status for each dimension using subscale scores. Verification with groups of children and adolescents indicated that the scale has good reliability, with Cronbach's α > 0.8 for the total score and subscales. In addition, the results of the confirmatory factor analysis showed that the scale has good construct validity. In this study, the scores provided by the respondent for all items were averaged, and the internal consistency of each measurement was acceptable (Cronbach's α = 0.951 to 0.964).

### 2.4. Data processing

Data management and statistical analysis were carried out using SPSS 25.0 and Mplus 8.1. Considering the research questions, we first performed descriptive statistics and calculated the correlation coefficient for each variable to examine the stability of PR and MHPs as well as the correlation between the two at different time points. Next, the latent growth model (LGM) (38) was used to separately establish unconditional linear LGMs for the three sets of follow-up data for PR and MHPs. On this basis, we investigated (1) the developmental trajectories of PR and MHPs, respectively, by constructing LGMs with parallel development; we examined (2) the influence of PR on the developmental trajectory of MHP; and finally, cross-lagged regression analysis was used to further confirm (3) the temporal order and bidirectional predictive effect between PR and MHPs. By combining the cross-lagged model with the latent growth model, we can examine the reciprocal relations between the variables. This relationship can be distinguished from the stricter causal effect (39). Additionally, we focus on the core problem that can be solved by the two models: whether there is developmental inertia or a reciprocating influence between variables in individuals after controlling for the stable inter-individual variation.

To avoid unnecessary model complexity associated with sample size, the model was tested using a manifest variable indicator (i.e., the mean score of the scale). The model fit was evaluated by using χ^2^, *df*, and CFI (>0.90), RMSEA (<0.08), and SRMR (<0.08) (40). When the model is saturated, that is, all the parameters to be estimated are equal to the elements in the covariance matrix with zero degrees of freedom, its fit index is no longer estimated, and only the path coefficient is considered (41). According to the data distribution in this study, the robust maximum likelihood estimation method was used to estimate the model parameters when establishing the LGM and the cross-lagged regression model (42).

### 2.5. Common method bias

To examine the extent to which the three sets of measurements were influenced by common method bias, the Harman's single-factor test was performed on the three sets of measurement data separately. The results showed that there is more than one factor with a characteristic root > 1 for each data set and that the variance explained by the first factor is 29.19, 35.59, and 35.83%, all less than the critical standard of 40% (43), indicating that this study was not significantly affected by common method bias.

## 3. Results

### 3.1. Descriptive statistics and correlation analysis of variables

The mean, standard deviation, and correlation coefficient matrix for PR and MHPs for each of the three sets of measurements are shown in Table 1. From T1 to T3, the PR level of the relocated adolescents showed an overall increasing trend, whereas the MHPs of the relocated adolescents showed an overall decreasing trend. In addition, there was a significant negative correlation between PR T1 and MHP T1, PR T2 and MHP T2, PR T3 and MHP T3 (*r* = −0.28 to −0.18, *p* < 0.01).

**Table 1:** The variable mean value, standard deviation and coefficient of correlation matrix.

**Variables**	**M(SD)**	**1**	**2**	**3**	**4**	**5**	**6**
1.PR T1	2.98 (0.92)	1					
2.PR T2	3.17 (0.80)	0.21^**^	1				
3.PR T3	3.30 (0.78)	0.11^**^	0.27^**^	1			
4.MHP T1	1.45 (0.54)	–0.18^**^	−0.13^**^	−0.10^**^	1		
5.MHP T2	1.43 (0.58)	−0.11^**^	–0.23^**^	−0.13^**^	0.28^**^	1	
6.MHP T3	1.39 (0.56)	−0.13^**^	−0.16^**^	−0.28^**^	0.27^**^	0.47^**^	1

PR, psychological resilience; MHP, mental health problems.

** *P* < 0.01.

### 3.2. Developmental trajectories of PR and MHPs

**Figure 1:**
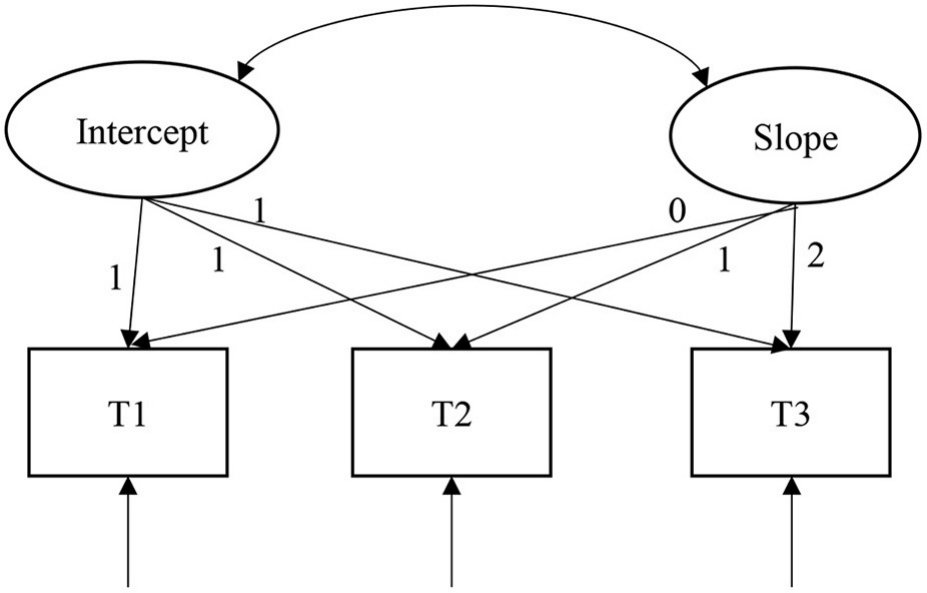
An unconditional linear latent growth model of psychological resilience or mental health problems.

The PR and MHP trends for relocated adolescents were examined by constructing unconditional linear LGMs (Figure 1). An unconditional linear LGM has two main parameters, intercept and slope. The intercept measures the initial levels of PR and MHP, with the factor loading fixed at 1 for the different measurement time points. The slope measures the rate and direction of linear changes in PR and MHPs, and because each measurement was separated by 1 year, the slope factor loadings for the three measurements are set to 0, 1, and 2.

As shown in Table 2, according to the unconditional linear LGM, the model fit of PR tends to saturate as follows: χ^2^(*df*) = 1.84(1), CFI = 0.99, RMSEA = 0.03, and SRMR = 0.01. The model fit of MHPs also tends to saturate: χ^2^ (*df*) = 0.26(1), CFI = 1.00, RMSEA = 0.00, and SRMR = 0.00. Thus, we only focus on their path coefficients. As seen in Table 3, in the unconditional linear LGM, the model intercept, i.e., the initial level of PR, is 2.99 (*p* < 0.01), and the initial level of MHP is 1.45 (*p* < 0.01), both of which are significantly > 0. The PR of the relocated adolescents showed an increasing trend (slope = 0.16, *p* < 0.01) and MHPs showed a linear decreasing trend (slope = −0.03, *p* < 0.01) in the three sets of measurements, thus supporting Hypothesis 1. In addition, the intercept variance (σ = 0.24, *p* < 0.01) and slope variance (σ = 0.08, *p* < 0.01) of PR each differ significantly, indicating that the initial level and rate of change in PR each differed systematically among individuals. The intercept variance (σ = 0.10, *p* < 0.01) and slope variance (σ = 0.04, *p* < 0.01) for MHPs are each significantly different, indicating that there were systematic differences in the initial level and rate of change in MHPs among individuals. Finally, the correlation between the intercept and slope of PR is significant (*r* = −0.55, *p* < 0.01), indicating that the higher the initial PR level, the slower the decline in PR over time; there was no significant correlation between the intercept and slope of mental health problems (*r* = −0.11, *p* > 0.05), indicating that the initial level of mental health problems was not related to the rate of change.

**Table 2:** Fit indices for linear latent growth models.

	**Model**	***χ^2^*(*df*)**	***p***	**CFI**	**RMSEA**	**SRMR**
PR	Unconditional Linear LGM	1.84 (1)	0.18	0.99	0.03	0.01
MHP	Unconditional Linear LGM	0.29 (1)	0.59	1.00	0.00	0.00
Parallel growth model		37.35 (7)	0.00	0.96	0.06	0.02
A cross-lagged regression model		31.71 (4)	0.00	0.96	0.07	0.03

PR, psychological resilience; MHP, mental health problems.

**Table 3:** Parameter estimates for unconditional latent growth models.

	**Intercept**		**Slope**		**Intercept and linear slope**
	**M**	**Variance**	**M**	**Variance**	**Correlation**
PR	2.99^**^	0.24^**^	0.16^**^	0.08^**^	−0.55^**^
MHP	1.45^**^	0.10^**^	−0.03^**^	0.04^**^	−0.11

*PR, psychological resilience; MHP, mental health problems*.

** *P* < 0.01.

### 3.3. Parallel growth model of PR to MHPs

**Figure 2:**
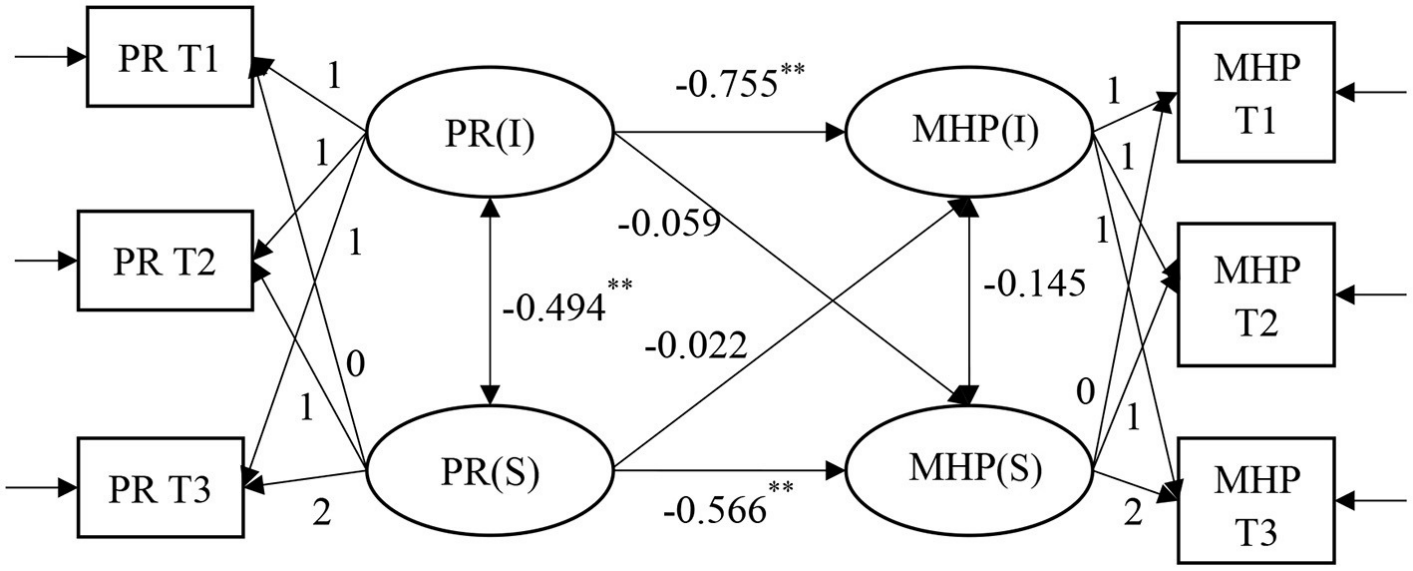
A parallel latent growth model of psychological resilience and mental health problems. The grade and mode of schooling of adolescents have been controlled. All parameter estimates in the figure are standardized results. ^**^P < 0.01.

To examine the relationship between the PR and MHPs of relocated adolescents, we developed a parallel growth model to simultaneously investigate the latent growth of PR and MHPs, and controlled the grade as a covariate. The intercept and slope in the PR model were used to predict the linear growth of MHPs; the results are shown in Figure 2. The fit indices of this model are shown in Table 2: χ^2^(*df*) = 37.35(7), CFI = 0.96, RMSEA = 0.06, and SRMR = 0.02, with good fit results.

The correlation coefficient between the intercept factor and slope factor of PR is −0.494 (*p* = 0.00), suggesting that the initial level of PR was related to the subsequent change rate. The regression coefficients from the PR intercept factor to MHPs intercept and slope factor were −0.755 (*p* = 0.00) and −0.059 (*p* = 0.64), respectively, indicating that the initial PR level had a significant negative predictive effect on the initial level of MHPs and no significant influence on the rate of change in MHPs, indicating that the lower the initial PR level, the higher the initial MHPs level. The regression coefficients of the slope factor of PR to the intercept and slope factors of MHPs are −0.022 (*p* = 0.88) and −0.566 (*p* = 0.00), respectively, indicating that the rate of change in PR had no significant predictive effect on the initial MHPs level and had a significant negative predictive effect on the rate of change in MHPs, indicating that the faster the decrease in PR, the faster the increase in MHPs. The above research results verify Hypothesis 2.

**Figure 3:**
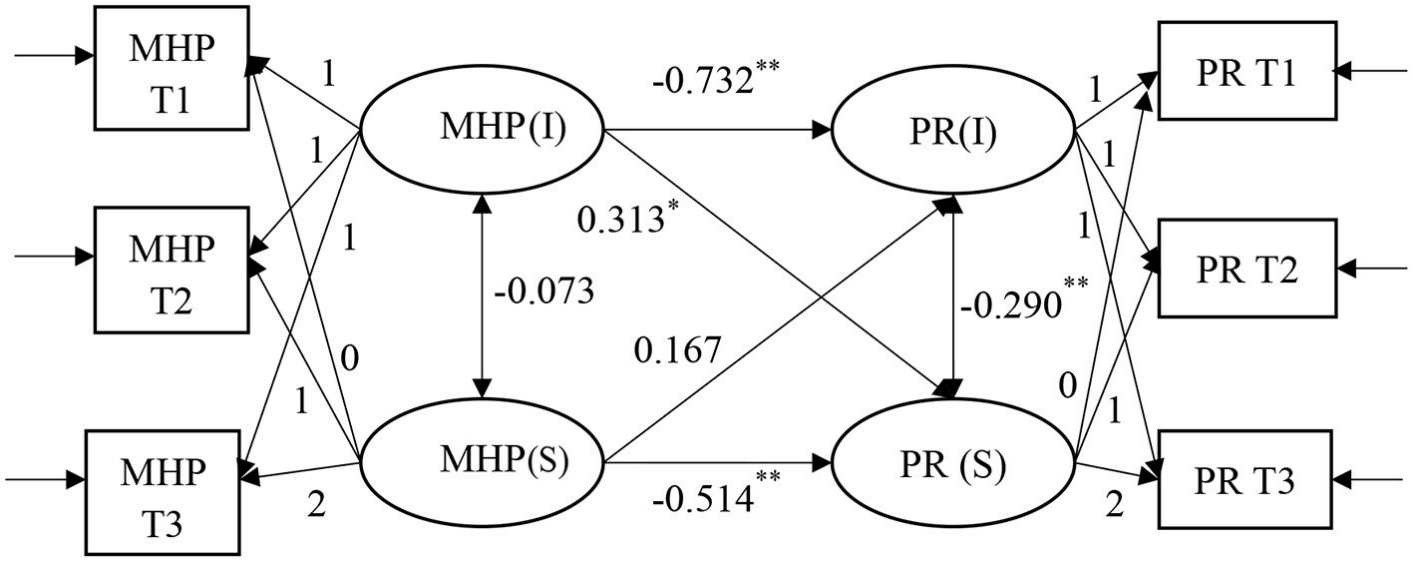
A competitive model of parallel latent growth model for mental health problems and psychological resilience. The grade and mode of schooling of adolescents have been controlled. All parameter estimates in the figure are standardized results. *P < 0.05, **P < 0.01.

To further test the causal relationship between PR and MHPs and whether there is mutual influence between the two and to avoid the inability to accurately capture the true relationship pattern between the two due to the predetermined assumptions, a competitive parallel LGM for MHPs predicting PR was developed by swapping the independent and dependent variables (Figure 3). The regression coefficients of the intercept factor of MHPs to the intercept and slope factors of PR are −0.732 (*p* = 0.00) and 0.313 (*p* = 0.04), respectively, indicating that the initial MHPs level had a significant negative predictive effect on the initial PR level and had a significant positive predictive effect on the change rate of PR. The regression coefficients of the slope factor of MHPs to the intercept and slope factors of PR are 0.167 (*p* = 0.329) and −0.514 (*p* = 0.00), respectively, indicating that the rate of change in MHPs had no significant predictive effect on the initial PR level and had a significant negative predictive effect on the rate of change in PR.

### 3.4. Cross-lagged regression analysis

An LGM helps to better capture the dynamic characteristics of the structure of interest. The above research results show a significant mutual negative predictive effect between the initial level and rate of change in PR and the initial level and rate of change in MHP. To further examine the interaction between the PR and MHPs of relocated adolescents over time and to strengthen the argument of the bidirectional effect, grade was controlled as a covariate, and cross-lagged regression analysis was conducted on the PR and MHPs of the relocated adolescents in the three measurements to obtain a more stable causal conclusion.

**Figure 4:**
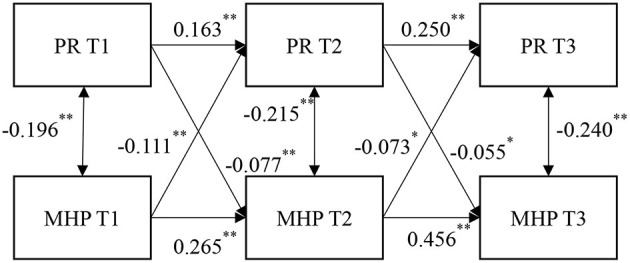
A cross-lagged regression model of mental health problems and psychological resilience. The grade and mode of schooling of adolescents have been controlled. All parameter estimates in the figure are standardized results. ^*^P < 0.05, ^**^P < 0.01.

The Cross-lagged regression model of PR and MHPs is shown in Figure 4, and the fit indices of this model are shown in Table 2: χ^2^*(df)* = 31.71(4), CFI = 0.96, RMSEA = 0.07, SRMR = 0.03, with good fit results. PR T1 significantly negatively predicted MHPs T2 (β = −0.077, SE = 0.027, *p* = 0.005), and PR T2 significantly negatively predicted MHPs T3 (β = −0.055, SE = 0.025, *p* = 0.029). MHPs T1 significantly negatively predicted PR T2 (β = −0.111, SE = 0.028, *p* = 0.000), and MHPs T2 negatively predicted PR T3 (β = −0.073, SE = 0.029, *p* = 0.012). The above research results and the findings in Section Cross-lagged regression analysis together verify Hypothesis 3.

## 4. Discussion

This study found that the PR of the relocated adolescents continuously improved across three surveys. The PR process varies with population, environment and risk exposure (20, 21). Overall, the main problems faced by the relocated people are weak employment competitiveness and weak livelihood. They are also facing changes in production and life-style, social communication style and social environment (44). This study takes the relocated adolescents as the research object. Their main contacts and sources of pressure are school and family. This group moved to the resettlement area in 2018. They had been living in the new resettlement area for 2 years since the first survey and had already begun to familiarize themselves with school and life. In addition, as the frequency of people going out during the epidemic has decreased, the relocation of young people to vulnerable areas has resulted in more contact with parents or elders at home. The interaction between the gradual adaptation to the school environment and the family's sense of familiarity and belonging has enhanced the adaptability and PR of the relocated adolescents. This finding also conforms to the compensation model of PR. This model considers that various environmental factors directly impact PR and combine with protective or promotive factors to improve the possibility of social adaptation, thus reducing the negative impact of risk (15, 45).

The study also found that the MHPs of the relocated adolescents decreased significantly across the three surveys. Family and school together constitute the growing environment of adolescents, which is why these two points often become the focus of research on adolescent MHPs. One study found that the school integration of relocated adolescents is better and is mutually integrated with the school learning environment, interpersonal relations and so on (46). This group also feels less discriminated against by others. Since the relocation policy will place adolescents in schools where the main source of students is the children of the relocated immigrants, they are not a minority of special groups in the school, and there are many peers have the same experiences. Therefore, a harmonious school environment may be a main reason for the reduction in the MHPs of relocated adolescents. The main way to transform the livelihood strategy of relocated people is to work outside (47). However, the relocated adolescents will suffer emotional loss and depression due to their parents' long-term work outside the home (48). During the epidemic, the time that relocated people spent working outside the home was reduced (49), which also increased the time that relocated adolescents spent communicating with their parents. This situation not only provides a stable family environment for the relocated adolescents but also tends to result in more stable mental health in this group.

This study constructed a parallel growth model and a cross-lagged model to verify the significant two-way and negative predictive effect between PR and MHPs of the relocated adolescents. Most studies have focused on the adaptation of relocated adolescents, and then explored their mental health based on adaptation (50, 51). In fact, when we regard PR as a process (14), it is equivalent to adaptation. Therefore, compared with the previous research on predicting MHPs by PR (or “adaptation”) of the relocated adolescents, our empirical results further prove that the improved mental health of the relocated adolescents is related to the improved PR. There is a chain effect: that is, MHPs affect PR, and PR further affects MHPs.

The results of this study are similar to those of other researchers in different populations (31). This agreement suggests that, at present, there are few studies on the psychological status quo and development of the relocated adolescents. However, due to the gradual promotion of the relocation policy, many relocated adolescents are still adapting to the new environment. If more targeted research findings can be produced, timely intervention can be carried out to improve the actual benefits of the “relocation of poverty alleviation in different places.”

This study has some limitations. From the perspective of statistical identification and clear insight into the research problem, an LGM requires at least three sets of measurements. However, the third time point could include factors that unintentionally distort the developmental trend; in such cases, a fourth set of measurement is needed to determine the developmental trend. Therefore, relevant studies in the future should take measurements at a minimum of four time points to better understand the development of PR and MHPs among relocated adolescents. Finally, data were collected through online surveys only.

## 5. Conclusion

The PR level of relocated adolescents increases over time, and MHPs decrease over time. The initial PR level of relocated adolescents has a negative predictive effect on the initial MHPs level, and the rate of change in PR has a negative predictive effect on the rate of change in MHPs. The PR and MHPs of relocated adolescents have a bidirectional, mutually influencing relationship.

## Data availability statement

The original contributions presented in the study are included in the article/supplementary material, further inquiries can be directed to the corresponding author.

## Ethics statement

The studies involving human participants were reviewed and approved by Ethics Review Committee of the State Key Laboratory of Cognitive Neuroscience and Learning at Beijing Normal University. Written informed consent to participate in this study was provided by the participants' legal guardian/next of kin.

## Author contributions

HZ conceived and designed the study. BW and XS contributed to data collection. JS and RZ analyzed the data. HZ, JS, and RZ wrote the paper. All authors reviewed and approved the manuscript.

## References

[1] Armstrong JM Ruttle PL Klein MH Essex MJ Benca RM. Associations of child insomnia, sleep movement, and their persistence with mental health symptoms in childhood and adolescence. Sleep. (2014) 37:901–9. 10.5665/sleep.3656 24790268 PMC3985117

[2] Hugh-Jones S Naidu J Al-Janabi H Bhola P Cooke P Fazel M. Safeguarding adolescent mental health in India (SAMA): study protocol for codesign and feasibility study of a school systems intervention targeting adolescent anxiety and depression in India. BMJ Open. (2022) 12:e054897. 10.1136/bmjopen-2021-054897 35379625 PMC8981280

[3] Fastame MC Mulas I Ruiu M. Associations between migration experience and perceived mental health in optimal ageing: evidence from the Sardinian Blue Zone. Int J Psychol. (2022) 57:271–8. 10.1002/ijop.12810 34549817 PMC9293095

[4] Chan ACY Piehler TF Ho GWK. Resilience and mental health during the COVID-19 pandemic: findings from Minnesota and Hong Kong. J Affect Disord. (2021) 295:771–80. 10.1016/j.jad.2021.08.144 34517251 PMC8422274

[5] Kotera Y Ting SH Neary S. Mental health of Malaysian University Students: UK comparison, and relationship between negative mental health attitudes, self-compassion, and resilience. High Educ. (2021) 81:403–19. 10.1007/s10734-020-00547-w

[6] Liang L-L Ren H Cao R-L Hu Y-Y Qin Z-Y Li C-E. The effect of COVID-19 on youth mental health. Psychiatr Q. (2020) 91:841–52. 10.1007/s11126-020-09744-3 32319041 PMC7173777

[7] Maunder RG Leszcz M Savage D Adam MA Peladeau N Romano D. Applying the lessons of SARS to pandemic influenza: an evidence-based approach to mitigating the stress experienced by healthcare workers. Can J Public Health. (2008) 99:486–8. 10.1007/BF03403782 19149392 PMC5148615

[8] Kavčič T Avsec A Zager Kocjan G. Psychological functioning of slovene adults during the COVID-19 pandemic: does resilience matter? Psychiatr Q. (2021) 92:207–16. 10.1007/s11126-020-09789-4 32556914 PMC7299145

[9] Masten AS. Resilience of children in disasters: a multisystem perspective. Int J Psychol. (2021) 56:1–11. 10.1002/ijop.12737 33325580

[10] Ye Q Su H. Policy practice and recapitalization: experience expression of anti-poverty by relocation of Guizhou Province. J China Agric Univ. (2016) 33:64–70. 10.13240/j.cnki.caujsse.2016.05.00720640187

[11] Zhao Y Zhang X. Effect of perceived discrimination on the psychological adaptation of children relocated to alleviate poverty: a longitudinal study. Chin J Sch Health. (2022) 43:100–3. 10.16835/j.cnki.1000-9817.2022.01.022

[12] Zhao Y Zhang X Guo J. Longitudinal study on the effect of family function on the psychological adaptation of children relocated for poverty alleviation. Chin J Health Psychol. (2022) 30:698–703. 10.13342/j.cnki.cjhp.2022.05.013

[13] Li Y Zhou Y Ru T Niu J He M Zhou G. How does the COVID-19 affect mental health and sleep among Chinese adolescents: a longitudinal follow-up study. Sleep Med. (2021) 85:246–58. 10.1016/j.sleep.2021.07.008 34388503 PMC8418314

[14] Ungar M Theron L. Resilience and mental health: how multisystemic processes contribute to positive outcomes. Lancet Psychiat. (2020) 7:441–8. 10.1016/S2215-0366(19)30434-1 31806473

[15] Gao Y-J Xie S-H Frost CJ. An ecological investigation of resilience among rural-urban migrant adolescents of low socioeconomic status families in China. J Community Psychol. (2020) 48:862–78. 10.1002/jcop.22303 31872898

[16] Ionescu T. Exploring the nature of cognitive flexibility. New Ideas Psychol. (2012) 30:190–200. 10.1016/j.newideapsych.2011.11.001

[17] Köber G Pooseh S Engen H Chmitorz A Kampa M Schick A. Individualizing deep dynamic models for psychological resilience data. Sci Rep. (2022) 12:8061. 10.1038/s41598-022-11650-6 35577829 PMC9110739

[18] Connor KM Davidson JRT. Development of a new resilience scale: the Connor-Davidson Resilience Scale (CD-RISC). Depress Anxiety. (2003) 18:76–82. 10.1002/da.10113 12964174

[19] Shen X Li Y-T Feng J Lu Z-X Tian K-M Gan Y. Current status and associated factors of psychological resilience among the Chinese residents during the coronavirus disease 2019 pandemic. Int J Soc Psychiatry. (2022) 68:34–43. 10.1177/0020764020980779 33300397

[20] Masten AS. Global perspectives on resilience in children and youth. Child Dev. (2014) 85:6–20. 10.1111/cdev.12205 24341286

[21] Ungar M Ghazinour M Richter J. Annual research review: what is resilience within the social ecology of human development? J Child Psychol Psychiatry. (2013) 54:348–66. 10.1111/jcpp.12025 23215898

[22] Gao Y-J Wong DSW. Strains and delinquency of migrant adolescents in China: an investigation from the perspective of general strain theory. Youth Soc. (2018) 50:506–28. 10.1177/0044118X15611308

[23] Orth Z van Wyk B. Adolescent mental wellness: a systematic review protocol of instruments measuring general mental health and well-being. BMJ Open. (2020) 10:e037237. 10.1136/bmjopen-2020-037237 32830115 PMC7445341

[24] Schoeps K Tamarit A De la Barrera U Lacomba-Trejo L Montoya-Castilla I del Rosario C. Social and psychological effects of COVID-19 pandemic on adolescents' and young adults' mental health: a cross-cultural mediation study. Psychol Rep. (2022) 1−28. 10.1177/00332941221100451 35531784 PMC9098395

[25] Tejerina-Arreal M Parker C Paget A Henley W Logan S Emond A. Child and adolescent mental health trajectories in relation to exclusion from school from the avon longitudinal study of parents and children. Child Adolesc Ment Health. (2020) 25:217–23. 10.1111/camh.12367 32516500 PMC7687195

[26] Qu X-Y Xie L-P Dai W Tang C-H Ma L-T. A study on the behavior and mental health of teenagers in relocation for poverty alleviation and relocation. Psychology Monthly. (2022) 17:18–9. 10.19738/j.cnki.psy.2022.01.007

[27] Wahlbeck K Cresswell-Smith J Haaramo P Parkkonen J. Interventions to mitigate the effects of poverty and inequality on mental health. Soc Psychiatry Psychiatr Epidemiol. (2017) 52:505–14. 10.1007/s00127-017-1370-4 28280872

[28] Johnson AK Hayes SN Sawchuk C Johnson MP Best PJ Gulati R. Analysis of posttraumatic stress disorder, depression, anxiety, and resiliency within the unique population of spontaneous coronary artery dissection survivors. J Am Heart Assoc. (2020) 9:e014372. 10.1161/JAHA.119.014372 32342736 PMC7428589

[29] Oducado RM Parreño-Lachica G Rabacal J. Personal resilience and its influence on COVID-19 stress, anxiety and fear among graduate students in the Philippines. Int J Educ Res Innov. (2021) 431–43. 10.46661/ijeri.5484

[30] Guilera G Pereda N Paños A Abad J. Assessing resilience in adolescence: the Spanish adaptation of the Adolescent Resilience Questionnaire. Health Qual Life Outcomes. (2015) 13:1–9. 10.1186/s12955-015-0259-8 26159814 PMC4498517

[31] Wu Y Sang Z-Q Zhang X-C Margraf J. The relationship between resilience and mental health in chinese college students: a longitudinal cross-lagged analysis. Front Psychol. (2020) 11:108. 10.3389/fpsyg.2020.00108 32116918 PMC7012791

[32] Shi W Zhao L Liu M Hong B-X Jiang L-H Jia P. Resilience and mental health: a longitudinal cohort study of Chinese adolescents before and during COVID-19. Front Psychiatry. (2022) 13:948036. 10.3389/fpsyt.2022.948036 36061276 PMC9428694

[33] Garagiola ER Lam Q Wachsmuth LS Tan TS Ghali S Asafo S. Adolescent Resilience during the COVID-19 Pandemic: A Review of the Impact of the Pandemic on Developmental Milestones. Behav Sci. (2022) 12:220. 10.3390/bs12070220 35877290 PMC9311591

[34] Hardiyati H Ahmad M Rahim R Musdalifah M. Adolescent resilience in facing the COVID-19 pandemic. Malays J Med Health Sci. (2022) 18:198–203.

[35] Hu Y-Q Gan Y-Q. Development and psychometric validity of the Resilience Scale for Chinese Adolescents. Acta Psychologica Sinica. (2008) 40:902–12. 10.3724/SP.J.1041.2008.00902

[36] Wang J-S Li Y He E-S. Development and standardization of mental health scale for middle school students in China. Soc Psychol. (1997) 0:15–20.

[37] Wang Z-Y. The Symptom checklist (SCL-90). Shanghai Archives of Psychiatry. (1984) 68–70.

[38] Bollen KA Curran PJ. Latent Curve Models: A Structural Equation Perspective. New York, NY: John Wiley & Sons, Ltd (2005). p. 1–293. 10.1002/0471746096

[39] Wen Z-L. Causal inference and analysis in empirical studies. J Psychol Sci. (2017) 40:200–8. 10.16719/j.cnki.1671-6981.20170130

[40] Kline RB. Principles and practice of structural equation modeling (3rd Edition). New York, NY: Guilford Press (2015).

[41] Steeger CM Gondoli DM. Mother-adolescent conflict as a mediator between adolescent problem behaviors and maternal psychological control. Dev Psychol. (2013) 49:804–14. 10.1037/a0028599 22612432 PMC4203150

[42] Dong Y Peng C-YJ. Principled missing data methods for researchers. SpringerPlus. (2013) 2:222. 10.1186/2193-1801-2-222 23853744 PMC3701793

[43] Podsakoff PM MacKenzie SB Lee J-Y Podsakoff NP. Common method biases in behavioral research: a critical review of the literature and recommended remedies. J Appl Psychol. (2003) 88:879–903. 10.1037/0021-9010.88.5.879 14516251

[44] Chen F-F Zhang Y-T Qiu H-G. How to ensure a smooth transition after “moving out of poor areas”? a study based on the employment governance of relocated households during the covid-19 pandemic. Comparative Economic & Social Systems. (2022) 48–59.

[45] Garmezy N Masten AS Tellegen A. The study of stress and competence in children: a building block for developmental psychopathology. Child Dev. (1984) 55:97–111. 10.2307/1129837 6705637

[46] Li M-P Liang F-M Yin C-Z. A study on the influence of discrimination perception on school integration of senior pupils in poverty alleviation and relocation. Psychological Monthly. (2022) 17:204–6. 10.19738/j.cnki.psy.2022.23.065

[47] Li C Qi Y-B He R-W. Progress and prospect of research on poverty alleviation relocation in china from the perspective of livelihood resilience. Geogr Inf Sci. (2022) 38: 74-81+129.

[48] Chen J. A practical study on social work intervention in the academic dilemma of left-behind children in relocation and relocation sites. Guizhou University, Guiyang, China. (2022).

[49] Xiao E-F. Investigation on the impact of COVID-19 epidemic on migrant workers in Hubei. Stat. Manag. (2021) 36:43–8. 10.16722/j.issn.1674-537x.2021.10.007

[50] Zhang L-Y Zuo Q-S Zhang Z-M Suo Y-D Luo M Tang J. Exploration on psychological reconstruction and social adaptation of adolescents in Xingyi city for poverty alleviation and relocation. Psychological Monthly. (2022) 17:206–8. 10.19738/j.cnki.psy.2022.17.067

[51] Zhao Y Wang P. A study on psychological adaptation of children relocated for poverty alleviation in inclusive education settings and the factors affecting it. Chin J Spec. Educ. (2021) 257:24–9.

